# Characteristics of ocular injuries associated with mortality in patients admitted with major trauma

**DOI:** 10.1186/s12886-024-03392-y

**Published:** 2024-03-19

**Authors:** Sruthi Kodali, Catherine H He, Sheel Patel, Alice Tao, Moshe Szlechter, Afshin Parsikia, Joyce N Mbekeani

**Affiliations:** 1 Montefiore Medical Center, Albert Einstein College of Medicine, Bronx, NY USA; 2 Department of Ophthalmology & Visual Sciences, Yale School of Medicine, New Haven, Conn USA; 3 Department of Ophthalmology, NYU Langone Health, New York, NY USA; 4 Department of Ophthalmology, Jamaica Hospital Medical Center, New York Medical College, Queens, NY USA; 5 Department of Surgery (Ophthalmology), Jacobi Medical Center, 1400 Pelham Parkway, Bronx, NY 10461 USA; 6 Department of Ophthalmology & Visual Sciences, Montefiore Medical Center/ Albert Einstein College of Medicine, Bronx, NY USA; 7 Department of Research Services, University of Pennsylvania, Philadelphia, PA USA

**Keywords:** Ocular trauma, Mortality, Firearms, Traumatic brain injury, Glasgow coma score, Injury severity score

## Abstract

**Background:**

Few ocular trauma studies have addressed mortality outcomes. We sought to determine characteristics of mortality-related ocular trauma admissions and compared them with non-fatal injuries.

**Methods:**

A retrospective study was conducted using de-identified data of patients admitted with major trauma from the National Trauma Data Bank (2008–2014). Patients with ocular injury were identified using ICD- 9CM codes. Demographics, intention and mechanism, types of ocular and head injuries, and injury severity were documented. Mortality was determined using post-admission disposition. Statistical analysis using student t-test, chi-square, and odds ratios (OR) calculations were performed with STATA-17 software. Significance was set at *P* < 0.05.

**Results:**

Of 316,485 patients admitted with ocular trauma, 12,233 (3.86%) were mortality related. Expired patients were older than survivors: mean (SD) of 50.1(25.5) vs. 41.5(22.8) years. White (OR = 1.32; *P* < 0.001), ≥ 65years old (OR = 2.25; *P* < 0.001), and male (OR = 1.05; *P* = 0.029) patients were most likely to expire than their counterparts. Common mechanisms of injury in survivors were falls (25.3%), motor vehicle traffic-occupant, MVTO (21.8%) and struck by/against (18.1%) and for fatal injuries, falls (29.7%), MVTO (21.9%) and firearms (11.5%). Traumatic brain injury (TBI) was documented in 88.2% of mortality-related admissions. Very severe injury severity scores (ISS > 24) (OR = 19.19; *P* < 0.001) and severe Glasgow Coma Score (GCS < 8) (OR = 19.22; *P* < 0.001) were most associated with mortality than survival. Firearms were most associated with very severe ISS (OR = 3.73; *P* < 0.001), severe GCS (OR = 4.68; *P* < 0.001) and mortality (OR = 5.21; *P* < 0.001) than other mechanisms. Patients with cut/pierce injuries had the greatest odds of survival (OR = 13.48; *P* < 0.001). Optic nerve/visual pathways injuries (3.1%) had the highest association with very severe ISS (OR = 2.51; *P* < 0.001), severe GCS (OR = 3.64; *P* < 0.001) and mortality (OR = 2.58; *P* < 0.001) than other ocular injuries. Black patients with very severe ISS (OR = 32.14; *P* < 0.001) and severe GCS (OR = 31.89; *P* < 0.001) were more likely to expire than other race/ethnicities with similar injury severity.

**Conclusions:**

Mortality-related admissions were older, male, and mostly of White race than ocular trauma admissions of survivors. Firearms were the deadliest mechanism. TBI was commonly associated and patients with optic nerve/pathway injuries, very severe ISS and severe GCS had higher mortality rates. Characteristics and demographic variations identified in this study may be useful in developing focused measures aimed at preventing trauma-related deaths.

**Supplementary Information:**

The online version contains supplementary material available at 10.1186/s12886-024-03392-y.

## Introduction

Ocular injury is the leading cause of preventable, monocular blindness and visual impairment in the United States [1]. The incidence of ocular injury is estimated at 28 per 100,000 person-years and as such is a significant public health concern [2]. Ocular injury accounts for a total of 2.4 million Emergency Department (ED) visits annually and comprises 38–52% of all eye-related ED visits [3, 4]. Given the prevalence of ocular injury and its association with poor visual outcomes, several studies have sought to characterize contributing epidemiological risk factors [5–7]. Risk factors associated with poorer visual outcomes following ocular injury include age greater than 60 years, injuries occurring outside of the workplace, gun-shot wounds, and trauma sustained during a fall [8]. 

Adverse visual outcomes have been linked to a significant decrease in quality of life and mental health [9]. Higher rates of anxiety and depression have been reported following injuries [10]. Several studies have associated visual impairment from multiple causes with increased mortality [11–13]. Although studies have reported the associations between ocular injury and non-fatal outcomes such as loss of vision, morbidity, decreased quality of life, and decline in mental health none, to our knowledge, have associated acute ocular injury and mortality.

Trauma is a leading cause of preventable mortality and traumatic brain injury is the single leading source of premature demise from trauma in the United States [14, 15]. The Centers for Disease Control and Prevention (CDC) has estimated that over 300 thousand deaths (92 per 100,000) result from injuries, with most resulting from falls, motor vehicle accidents and firearms trauma [16]. In a previous epidemiologic study, we observed an incidence of deaths of 12,233 (3.9%) among patients admitted for major trauma with ocular injury in the United States [17]. Although the common mechanisms of injury were different from those outlined by the CDC, we found that a substantial number of patients (184,124, 58,2%) were documented to have TBI. This prompted our interest in identifying risk factors in acute trauma with ocular injuries that were associated with mortality.

To this end, we sought to identify and compare risk factors for non-fatal and fatal injuries in patients admitted with major trauma and ocular injuries, using the same NTDB database. Findings from this study may help identify at risk patients for appropriate triaging and management planning. With confirmation, our results may also provide a foundation for the development of public health strategies that help reduce trauma-associated mortality.

## Methods and participants

This retrospective study used data from the National Trauma Data Bank (NTDB) collected between the years 2008–2014 and was approved by the institutional review board (IRB) of Albert Einstein College of Medicine, Bronx, New York. All methods were carried out in accordance with relevant guidelines and regulations of the Declaration of Helsinki. The NTDB is the largest national registry containing de-identified data of hospitalized trauma cases and was established by the American College of Surgeons (ACS) [18]. Data from the NTDB can be acquired upon request from the ACS.

In this study, we evaluated patients who were admitted with ocular trauma. Eligible patients were identified using International Classification of Diseases, Ninth Revision, Clinical Modification (ICD-9-CM) diagnosis and procedure codes as well as external causes of injury (E-codes). The ICD-9-CM codes can be found in a previous study by He et al. [17] that utilized the same codes from the same data source. The following variables were documented for each patient that met inclusion criteria: age, sex, race, ethnicity, health insurance coverage, systolic blood pressure at admission, presence of diabetes and/or hypertension, types of ocular injury, intent of injury, location where injury took place, related intracranial injuries, length of hospital admission, and level of hospital trauma service (Levels I-IV: both ACS-verified and state-designated centers). Types of ocular injury were grouped into categories based on the type and location of the injury as previously described. [17] Patients were also evaluated for presence of traumatic brain injury (TBI). TBI was identified based on Center for Disease Control criteria of ICD-9 CM codes for head injuries including extradural hemorrhage, cerebral contusion, base of skull fracture, intracerebral hemorrhage, vault skull fracture, subarachnoid hemorrhage, subdural hemorrhage, and fracture of the facial bone [18]. Details of the ICD-9 CM codes used are outlined in a previous study [19]. 

Glasgow Coma Score (GCS) and injury severity score values were also documented for each patient. GCS is a numerical index of degrees of traumatic brain injury and is documented in all trauma patients by Emergency Medical Services and upon admission to the emergency departments [18]. It ranges from 3 to 15 with lower numbers reflecting more traumatic brain injury. The injury severity score (ISS) assigns a numerical score as a measure of the degree of global injury severity. It ranges from 1 to 75 which represents a range from minor injuries (designated by lower scores) to more severe injuries resulting in an increased risk of mortality (designated by higher scores). Location and region, based on US census regions (Northeast, West, Midwest, and South) were documented as well. Post-admission dispositions for each patient were recorded, including home discharge, transfer to a nursing home, transfer to rehabilitation facility, transfer to another hospital, transfer to hospice services, left against medical advice and death on discharge. The dispositions were used to determine patient mortality.

### Statistical analysis

The statistical analysis and presentation of findings followed the STROBE (Strengthening the Reporting of Observational Studies in Epidemiology) specifications for reporting epidemiologic studies. The mean (SD) and median (interquartile range [IQR]) were calculated for all continuous variables. Values for these variables were grouped into categories for analysis. The population was stratified by age groups of less than or equal to 20years (pediatric age), 21 through 64years (working age), and greater than or equal to 65years (older age). Injury severity scores (ISS) were categorized according to NTDB subclassifications of minor (ISS = 1–8), moderate (ISS = 9–15), severe (ISS = 16–24), and very severe (ISS > 24). Glasgow Coma Scores (GCS) were categorized into mild (GCS = 13–15), moderate (GCS = 9–12), and severe (GCS ≤ 8). Comparative analysis of relative associations with variables was conducted for the major race/ethnicities of White, Black, Asian, and Hispanic patients. Characteristics of survivors were compared with patients who expired.

Categorized data was analyzed to detect associations between variables, using paired, two-tailed t-test and χ^2^ (chi-squared) tests. Univariate logistic regression analysis was conducted to identify relative associations, which were expressed in odds ratios (OR), 95% confidence intervals (CI), and two-tailed P-values. Statistical significance was set at *P* < 0.05. Descriptive and analytical calculations were performed using STATA, version 17 (StataCorp, College Station, TX). Graphs and tables were constructed using Microsoft Excel and Word (Microsoft Corp., Redmond, WA). Cases with data points documented as “unknown “or “undetermined” were excluded from analysis.

## Results

### General characteristics in mortality-related injuries

**Fig. 1:**
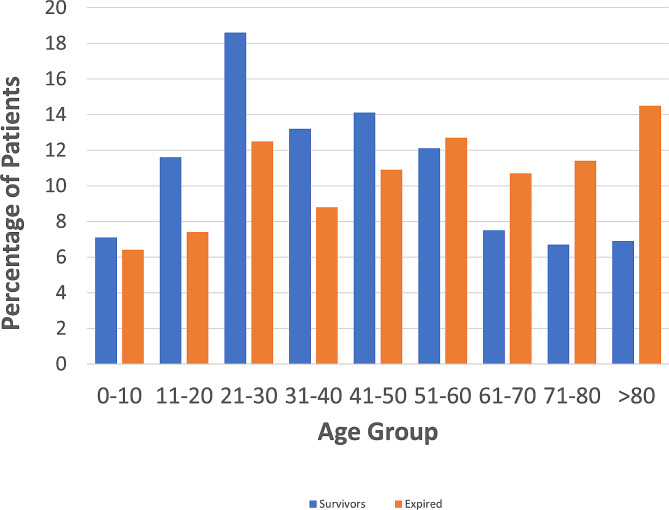
Percentage of ocular trauma in survivors and expired in different age groups - National Trauma Data Bank (2008–2014). **Legend**: Patients between 21 to 64year were the largest age strata with 6091 (49.8%) cases

**Fig. 2:**
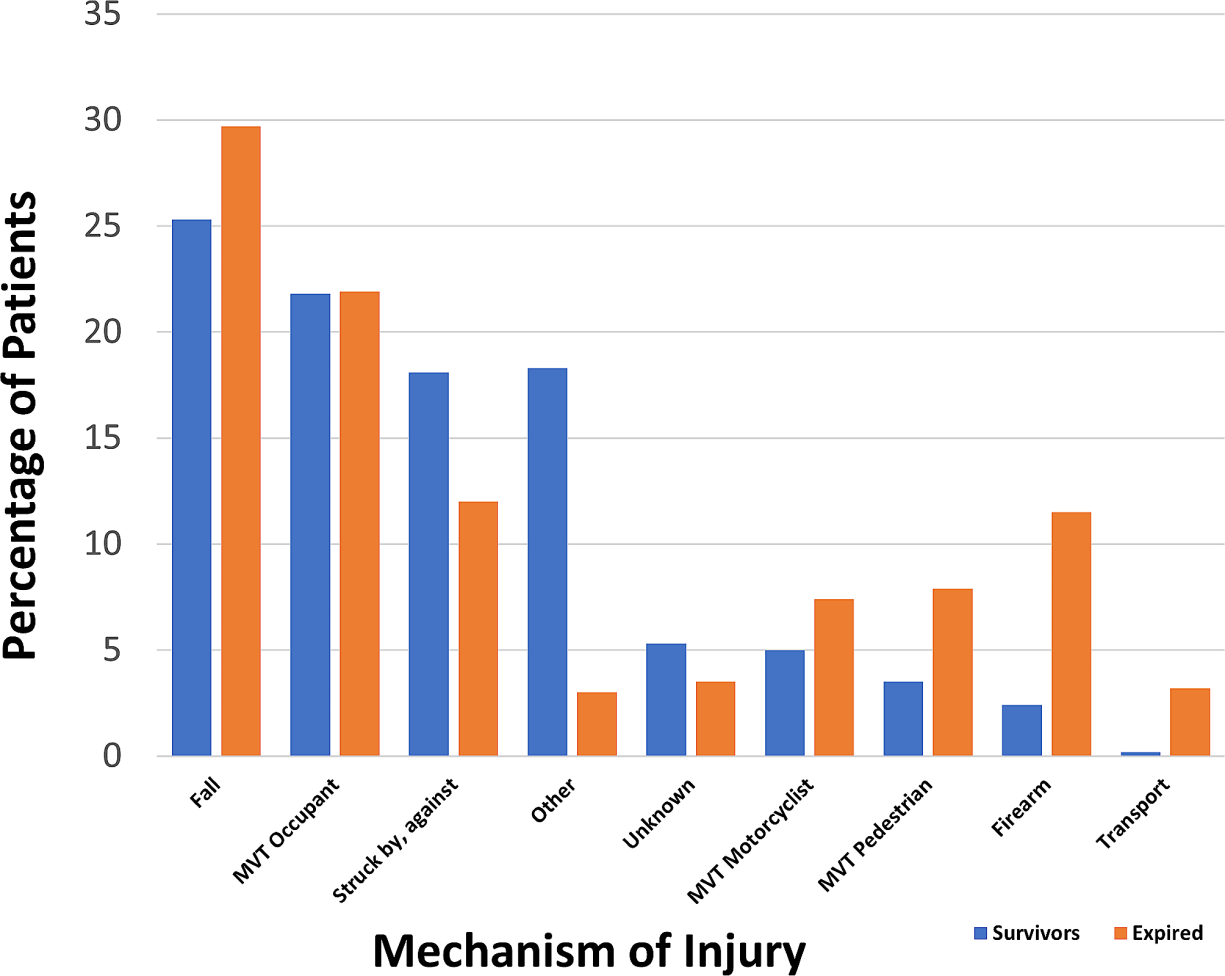
Mechanisms of Injury in Non-Fatal and Mortality-Related Ocular Trauma. **Legend**: Frequent mechanisms for expired patients were falls (29.7%), motor vehicle trauma-occupant (MVTO) (21.9%) and firearms (11.5%)

Between 2008 and 2014, 12,233 (3.86%) of 316,485 patients admitted with ocular injuries had documented mortality-related trauma. Of these patients, 8598 (70.3%) were male. The mean (SD) age was 50.1 (25.5) years. Patients between 21 and 64 year were the largest age strata with 6091 (49.8%) cases (Fig. 1). However, patients ≥ 65years old admitted with trauma were most likely to succumb to their injuries than other age groups (see below for analysis). The common races affected were White (71.8%), Black (10.8%), and Asian (2.2%). 17.4% were documented as “other” race. 1254 (10.3%) identified as Hispanic. Frequent mechanisms were falls (29.7%), motor vehicle trauma-occupant (MVTO) (21.9%) and firearms (11.5%) (Fig. 2). Common ocular injuries amongst those who expired were contusions of the eye/adnexa (49.1%) and orbital fractures (30.1%). Traumatic brain injury (TBI) was documented in 10,787 patients (88.2%) and frequent associated head injuries were facial fractures (61.3%) and subdural hemorrhages (35.4%). Mean (SD) injury severity score was 29.8 (13.4) and Glasgow coma score was 6.5(4.8). Most injuries were unintentional (75.9%) and occurred in street (44.6%) and home (34.1%) locations, and in the Southern US census region (37.7%). Table 1 provides a summary of the descriptive epidemiologic characteristics for fatal and non-fatal major trauma with ocular injuries.

**Table 1:** Descriptive characteristics of patients with mortality-related ocular trauma (survivors vs. expired), National Trauma Data Bank (2008–2014)

Characteristic	No. Survived (%), *n* = 304,252	No. Expired (%), *n* = 12,233	Characteristic	No. Survived (%), *n* = 304,252	No. Expired (%), *n* = 12,233
**Year**			**Age (years)**		
2008	35,662 (11.7%)	1556 (12.7%)	Mean (SD)	41.5 (22.8)	50.1 (25.5)
2009	39,923 (13.1%)	1660 (13.6%)	0–10	21,753 (7.1%)	778 (6.4%)
2010	41,526 (13.6%)	1518 (12.4%)	11–20	35,325 (11.6%)	909 (7.4%)
2011	24,376 (13.9%)	1706 (13.9%)	21–30	56,506 (18.6%)	1535 (12.5%)
2012	47,059 (15.5%)	1982 (16.2%)	31–40	40,276 (13.2%)	1077 (8.8%)
2013	46,840 (15.4%)	1786 (14.6%)	41–50	42,831 (14.1%)	1332 (10.9%)
2014	50,866 (16.7%)	2025 (16.6%)	51–60	36,719 (12.1%)	1549 (12.7%)
			61–70	22,926 (7.5%)	1307 (10.7%)
**Gender**			71–80	20,395 (6.7%)	1398 (11.4%)
Male	211,026 (69.4%)	8598 (70.3%)	> 80	21,118 (6.9%)	1779 (14.5%)
Female	93,226 (30.6%)	3635 (29.7%)	Unknown	6403 (2.1%)	569 (4.7%)
**Race**			**Length of Stay**		
Black	46,341 (15.2%)	1319 (10.8%)	Mean (SD)	6.2 (10.0)	6.4 (10.6)
White	200,329 (65.8%)	8786 (71.8%)	1 day	75,396 (24.8%)	3711 (30.3%)
Native American	3187 (1%)	67 (0.5%)	2–3 days	92,141 (30.3%)	2961 (24.2%)
Asian	5115 (1.7%)	268 (2.2%)	4–6 days	60,874 (20%)	2026 (16.6%)
Hawaiian/Pacific Islander	682 (0.2%)	30 (0.2%)	Greater than 6	75,282 (24.7%)	3527 (28.8%)
Other	48,598 (16.1%)	2128 (17.4%)	Unknown	559 (0.2%)	8 (0.1%)
**Ethnicity**			**Injury Severity Score**		
Hispanic	37,630 (12.4%)	1254 (10.3%)	1–8 (minor)	118,084 (38.8%)	417 (3.4%)
			9–15 (moderate)	85,856 (28.2%)	1104 (9%)
**Mechanism**			16–24 (Severe)	54,688 (18%)	1888 (15.4%)
Fall	77,048 (25.3%)	3630 (29.7%)	> 24 (very severe)	32,436 (10.7%)	8208 (67.1%)
MVT Occupant	66,293 (21.8%)	2673 (21.9%)	Unknown	13,188 (4.3%)	616 (5%)
Struck by, against	55,214 (18.1%)	366 (3%)			
MVT Motorcyclist	15,248 (5%)	900 (7.4%)	**Ocular Injuries**		
MVT Pedestrian	10,533 (3.5%)	967 (7.9%)	Open wound of ocular adnexa	72,448 (23.8%)	1515 (12.4%)
Other	55,663 (18.3%)	1462 (12%)	Open wound of eyeball	28,831 (9.5%)	1074 (8.8%)
Unknown	16,246 (5.3%)	429 (3.5%)	Superficial injury	33,979 (11.2%)	1077 (8.8%)
Transport, other	701 (0.2%)	397 (3.2%)	Contusion of eye/ adnexa	102,456 (33.7%)	5126 (41.9%)
Firearm	7306 (2.4%)	1409 (11.5%)	Foreign body on external eye	614 (0.2%)	6 (< 0.1%)
**Intention of Injury**			Burn confined to eye and adnexa	2871 (0.9%)	97 (0.8%)
Assault	61,109 (20.1%)	1486 (12.1%)	Injury to optic nerve and pathways	3671 (1.2%)	374 (3.1%)
Self-inflicted	3058 (1%)	897 (7.3%)	Injury to other cranial nerves (III, IV, VI)	6878 (2.3%)	145 (1.2%)
Unintentional	222,356 (73.1%)	9282 (75.9%)	Orbital fracture no other facial fracture	120,105 (39.5%)	4782 (39.1%)
Other	122 (< 0.1%)	4 (< 1%)	Hyphema	59 (< 0.1%)	0 (0%)
Undetermined	1361 (0.4%)	135 (1.1%)			
Unknown	16,246 (5.3%)	429 (3.5%)	**Locations of Injury**		
			Farm	1697 (0.6%)	56 (0.5%)
**Insurance**			Home	81,272 (26.7%)	4170 (34.1%)
Commercial	102,252 (33.6%)	3466 (28.3%)	Industry	6275 (2.1%)	169 (1.4%)
Medicare	52,306 (17.2%)	3300 (27%)	Mine	157 (0.1%)	5 (< 0.1%)
Medicaid	43,530 (14.3%)	1513 (12.4%)	Public building	16,853 (5.5%)	470 (3.8%)
Self-pay	53,589 (17.6%)	1887 (15.4%)	Recreation	11,989 (3.9%)	166 (1.4%)
Other government	10,084 (3.3%)	313 (2.6%)	Residential Institution	10,449 (3.4%)	498 (4.1%)
Not billed	2489 (0.8%)	104 (0.9%)	Street	123,700 (40.7%)	5454 (44.6%)
Unknown	40,002 (13.2%)	1095 (9%)	Other	15,451 (5.1%)	446 (3.6%)
			Unspecified	27,067 (8.9%)	508 (4.2%)
			Unknown	9342 (3.1%)	291 (2.4%)

### Comparative analysis

#### Demographic differences

Logistic regression analysis revealed statistically significant demographic differences between those who survived and those who expired. Expired patients were older than survivors: mean (SD) of 50.1 (25.5) vs. 41.5 (22.8) years. Patients most likely to expire were those ≥ 65 years old (OR = 2.25, CI 2.16–2.34; *P* < 0.001) whereas those most likely to survive were between 21 and 64 years old (OR = 1.53, CI 1.48–1.59; *P* < 0.001). Males (OR = 1.05, CI 1.00-1.09; *P* = 0.029) were most likely to succumb to their injuries than female patients. These males were also younger than females with mean (SD) age of 48.6(23.9) vs. 53.7(28.8) years; (*P* < 0.001). With respect to race/ethnicity, White patients were more likely to die than Black and Hispanic patients (OR = 1.32, CI 1.27–1.38; *P* < 0.001). However, Black (OR = 1.30, CI 1.15–1.47; *P* < 0.001) and Hispanic patients (OR = 1.24, CI 1.09–1.40; *P* < 0.001) were similarly more likely to die within a day of admission, while White patients were more likely to survive until 4-6days of admission (OR = 1.30, CI 1.16–1.46; *P* < 0.001). Further demographic differences in mechanism, intention and severity of injury are detailed below.

### Ocular injuries and head trauma

88.2% of expired patients suffered TBI compared to 57.0% of survivors. Patients with optic nerve/visual pathways injuries comprised only 3.1% of ocular injuries and had the highest odds of having documented concomitant TBI (OR = 15.51, CI 5.25–75.68; *P* < 0.001). These patients had the highest odds of associated base of skull fractures (OR = 1.67, CI 1.32–2.11; *P* < 0.001), and very severe ISS > 24 (OR = 2.51, CI 1.83–3.49; *P* < 0.001), and severe GCS < 8 (OR = 3.64, CI 2.51–5.44; *P* < 0.001). Consequently, patients with optic nerve/visual pathways injuries had the highest odds of mortality (OR = 2.58, CI = 2.32, 2.88; *P* < 0.001) than other ocular injuries. Indeed, they were most likely to expire within 3 days of admission than other ocular injuries (OR = 1.65, CI 1.30–1.08; *P* < 0.001). Of patients admitted with TBI, those with traumatic optic neuropathy were most associated with mortality (OR = 2.27; CI, 2.02–2.55; *P* < 0.001) than other visual pathway injuries. In comparison, patients who suffered open wound of the ocular adnexa had the highest odds of survival (OR = 2.21, CI 2.09–2.34; *P* < 0.001).

Regarding associated head injuries, patients with intracerebral hemorrhages had the highest odds of mortality (OR = 6.59, CI 6.31–6.88; *P* < 0.001), followed by those with base of the skull fractures (OR = 5.78, CI 5.53–6.04; *P* < 0.001), subdural hemorrhage (OR = 5.34, CI 5.14–5.56; *P* < 0.001), subarachnoid hemorrhage (OR = 4.87, CI 4.68–5.06; *P* < 0.001). Patients with associated base of skull (OR = 1.68, CI 1.54–1.83; *P* < 0.001) and skull vault fractures (OR = 1.66, CI 1.52–1.80; *P* < 0.001) had similarly greater odds of expiring within a day of admission than patients with other head trauma. Patients with facial fractures were most likely to survive their injuries (OR = 0.85, CI 0.82–0.89, *P* < 0.001) and those that died were more likely to survive greater than 6 days after admission, than patients with other head injuries (OR = 1.25, CI 1.15–1.35; *P* < 0.001) (Supplemental Table 1).

### Intent and mechanism of injury

Most mortality-related injuries were due to blunt trauma in both survivors (82.8%) and patients who expired (76.7%). In both groups, unintentional injury was the most common intent of injury (Table 1). Assault victims were most likely to survive (OR = 1.87, CI 1.77–1.98; *P* < 0.001) than those of other intentions. Patients admitted with self-inflicted injury were most likely to expire (OR = 7.66, CI 7.10–8.28; *P* < 0.001) than those who suffered injuries from other intentions. There were also distinct differences between age groups and sex. Patients ≥ 65 years old were most likely to suffer unintentional injury (OR = 5.90, CI 5.13–6.76; *P* < 0.001) whereas patients < 20 years old were most likely to be injured through assault (OR = 6.54, CI 5.78–7.40; *P* < 0.001). Patients between 21-64years old had the greatest odds of expiring from self-inflicted injuries (OR = 2.51, CI 2.16–2.91; *P* < 0.001). Males were most likely to expire following self-inflicted injury (OR = 2.69, CI 2.21–3.26; *P* < 0.001) while females died after unintentional injuries (OR = 1.71, CI = 1.54–1.90; *P* < 0.001) than other intentions. Regrading race, Black (OR = 5.41, CI 4.74–6.19; *P* < 0.001) and Hispanic (OR = 2.29, CI 1.97–2.66; *P* < 0.001) patients were most likely to be injured and expire following assault injuries. White patients were most likely to be of unintentional (OR = 2.30, CI 2.09–2.53; *P* < 0.001) and self-inflicted injuries (OR = 1.88, CI 1.57–2.26; *P* < 0.001) than other race/ethnicities. Asian patients did not exhibit a strength of association with any intention (Supplemental Table 2).

**Fig. 3:**
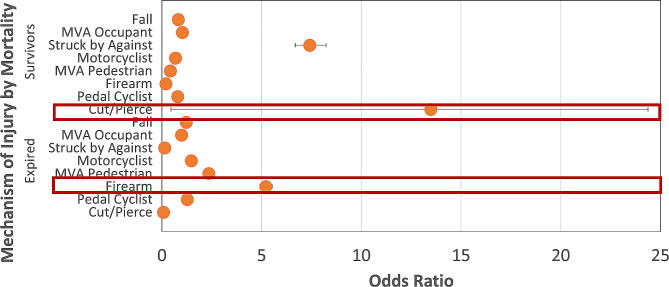
Logistic Regression Analysis of Association Between Mortality and Mechanism of Injury in Ocular Trauma. **Legend**: Cut/Pierce mechanism was most associated with survival while firearms were most associated with mortality

Differences were observed in mechanisms of injury. Common mechanisms in survivors were falls (25.3%), motor vehicle traffic occupant accidents (MVTO) (21.8%) and struck by/against (SBA (18.1%). For fatalities, falls were also the most common (29.7%) mechanism, followed by MVTO (21.9%) and firearms (11.5%) (Fig. 2). Firearm injuries had the greatest odds of fatality (OR = 5.21, CI 4.90–5.53; *P* < 0.001) than other mechanisms while victims of cut/pierce injuries had the greatest odds of survival (OR = 13.48, CI 7.46–24.37; *P* < 0.001) (Fig. 3). Additionally, patients admitted with firearms-related injuries were more likely to expire within a day of admission (OR = 3.19, CI 2.85–3.59; *P* < 0.001) while victims of struck by/against (OR = 1.52, CI 1.21–1.89; *P* < 0.001) and falls (OR = 1.46, CI 1.46, CI 1.34–1.59; *P* < 0.001) survived greater than six days before their demise. When classified by race/ethnicity, White patients were most likely to experience mortality-related injury due to falls (OR = 2.32, CI 2.10–2.56; *P* < 0.001), whereas Black patients had the highest odds of firearm injury (OR = 2.73, CI 2.36–3.16; *P* < 0.001). Both Asian (OR = 2.08, CI 1.43–2.95; *P* < 0.001) and Hispanic patients (OR = 1.70, CI 1.40–2.05; *P* < 0.001) were most likely to be victims of MVT-pedestrian trauma.

**Fig. 4:**
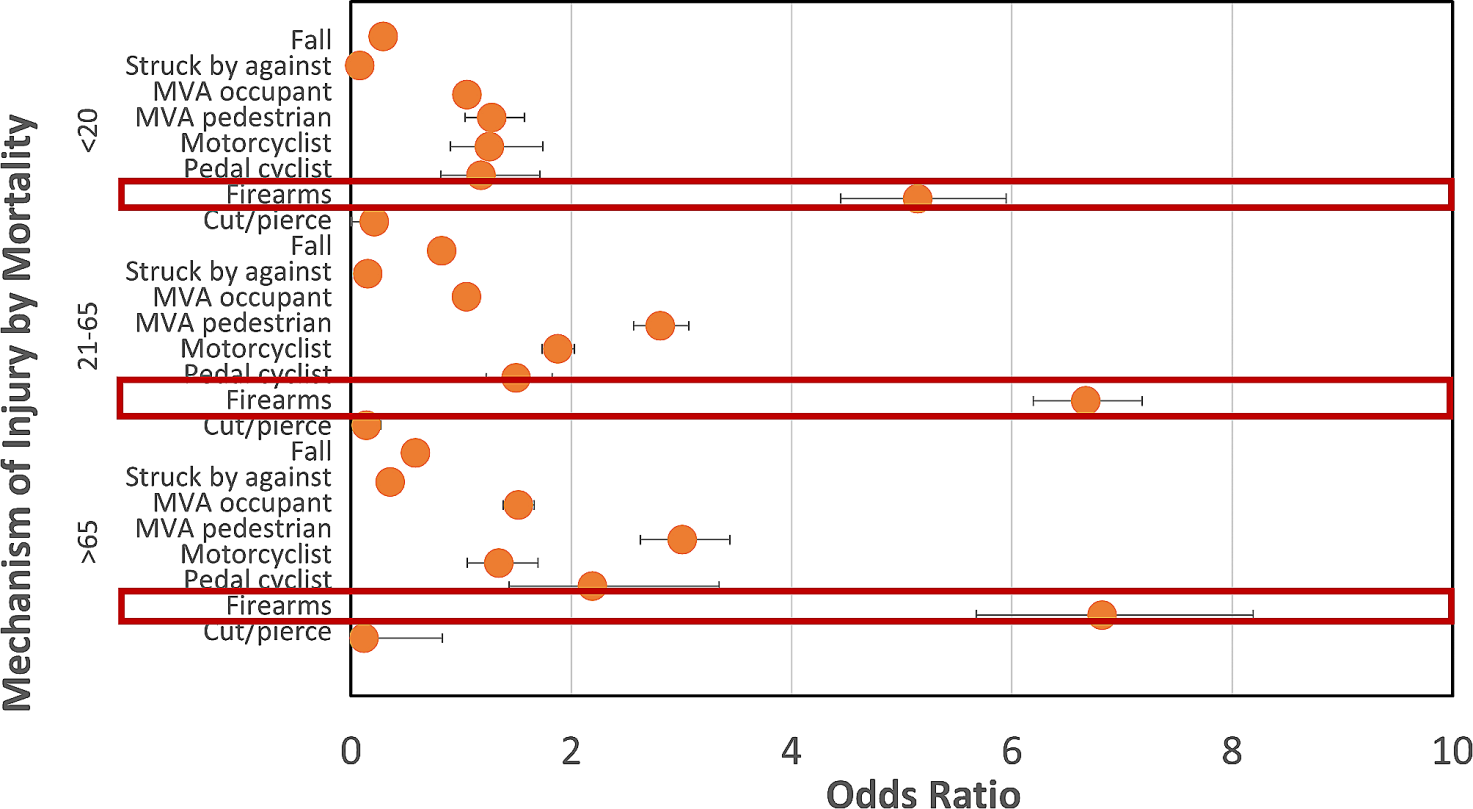
Logistic Regression Analysis of Association Between Age and Mechanism of Injury in Mortality Related Ocular Trauma. **Legend**: Firearms were most associated with mortality in all age groups

Regarding sex, admitted males were most likely to expire following MVT-motorcycle (OR = 4.05, CI = 3.25–5.10; *P* < 0.001). Females were mostly victims of falls (OR = 2.28, CI 2.00-2.37; *P* < 0.001). Comparison by age also yielded differences in mortality-associated mechanisms of injury. Firearm injuries had the greatest odds of mortality compared to other mechanisms across all age groups, with the highest odds in those ≥ 65 years old (OR = 6.82, CI 5.67–8.19; *P* < 0.001) and lowest odds in those ≤ 20 years (OR = 5.14, CI 4.45–5.95; *p* < 0.001) (Fig. 4). Patients ≤ 20 years old had greatest odds of cut/pierce injury (OR = 3.28, CI 3.07–3.51; *P* < 0.001) and were most likely to survive these injuries (OR = 48.12, CI 6.78-342.43; *P* < 0.001). Although falls were the most common mechanism of both fatal and non-fatal trauma, those ≤ 20 years old had greater odds of survival after falling (OR = 3.43, CI 2.59–4.54; *P* < 0.001) and those 21–64 years old had the lowest odds of survival after falling (OR = 1.21, CI 1.12–1.31; *P* < 0.001), followed by patients ≥ 65 years old (OR = 1.70, CI 1.59–1.82; *P* < 0.001).

### Severity of injury

Very severe injury severity scores (ISS > 24) (OR = 19.20, CI 18.42–20.01; *P* < 0.001) and severe GCS (< 8) (OR = 19.22, CI 18.43–20.04; *P* < 001) were more associated with mortality. Patients with very severe ISS were more likely to die if ≤ 20 years old (OR = 42.15, CI 36.75–48.25, *P* < 0.001), male (OR = 20.73, CI 19.70-21.81, *P* < 0.001), and of Black race (OR = 32.14, CI 28.17–36.67, *P* < 0.001). Similarly, patients with severe GCS were also most likely to perish if they were ≤ 20 years (OR = 135.26, CI 106.94-171.07; *P* < 0.001), male (OR = 20.33, CI 19.31–21.42, *p* < 0.001), or of Black race (OR = 31.89, CI 27.74–36.67, *P* < 0.001) (Supplemental Tables 3 and 4). Firearms were most associated with very severe (OR = 3.73, CI 3.56–3.91, *P* < 0.001) and severe traumatic brain injury (OR = 4.68, CI 4,47-4.89, *P* < 0.001).

Comparing insurance coverage, patients that were covered by Medicare were most likely to expire (OR = 1.82, CI 1.75–1.90, *P* < 0.001) and had higher odds of mortality compared to patients with commercial insurance (OR = 0.78, CI 0.75–0.81 *P* < 0.001). Indeed, patients covered by commercial insurance (OR = 1.28, CI 1.23–1.34, *P* < 0.001) had the highest odds of survival.

## Discussion

### Summary of findings

Most mortality-related studies in ophthalmology have evaluated different levels of diabetic retinopathy with all-cause deaths [20–22] and few publications have reported associations between visual disability and premature mortality [11–13]. Although mortality has been reported in several ocular trauma papers, to our knowledge, known have sought to identify variables most related to early demise following trauma. Our study found that amongst patients admitted with major trauma with ocular injuries, those most likely to be victims of trauma-related mortality were older than 65years, male and White patients. The majority had associated traumatic brain injury. Ocular and head injuries most associated with mortality were optic nerve/visual pathway injuries and base of skull fractures. Common mechanisms were falls, MVTO injuries in non-fatal and fatal injuries. However, the fatal injury category had substantially more firearms injuries than the survivors. Firearms were most associated with very severe injury severity score, and severe traumatic brain injury, and thus mortality, while cut/piece injuries had the lowest association with fatality. Although falls were the most common mechanism of both fatal and non-fatal trauma, those ≤ 20yrs had greater odds of survival after falling. Both very severe ISS and severe GCS were more associated with mortality than survival. However, Black patients with high levels of injury severity were more likely to die than other races/ethnicities in the same strata.

### Ocular injuries and mortality

We established those patients with higher degrees of injury severity (GCS < 8, ISS > 24) were most associated with mortality than patients with less severe injury scores. Consequently, visual pathway injuries were associated with more severe presentations and had increased odds of mortality than other ocular injuries. In contrast, patients who had open wounds of ocular adnexa were associated with less severe pathologies (GCS 13–15) and higher rates of survival. Clearly injuries to the optic nerve and visual pathways can be categorized as TBI, the number one cause of death from trauma [14, 15]. Traumatic brain injury has received most attention in relation to sport and combat related injury. However, it is now increasingly recognized in other forms of trauma. Numerous studies have examined visual and ocular sequelae of traumatic brain injury (TBI) [23–29]. However, few have evaluated the concurrence of ocular trauma and TBI and post-admission mortality. We found the rate of TBI in all 316,845 patients admitted with major trauma and ocular injuries was less (58.2%) than for those who succumbed to their injuries (88.2%). Gise et al. [30] evaluated visual pathway injuries in pediatric patients and found that this group had a higher mortality (17.6%) compared to all pediatric patients admitted with major trauma and ocular injuries (2.9%). Our findings comport with this mortality data and underscore the impact of TBI and visual pathway injuries post-admission hospital mortality.

Using the same NTDB database, during this time, it was noted that traumatic optic neuropathy was the most common visual pathway injury [17, 30]. The optic nerves are vulnerable to direct injuries through avulsion, transection, contusion, optic nerve sheath and orbital hemorrhages. Indirect impact of rapid acceleration/deceleration injuries resulting in transmitted traction and shearing forces are more common than direct injury. These injuries occur most commonly in the intracanalicular section at the skull base [31]. This supports our findings that base of skull fractures have higher mortality rates than most other head injuries (Supplemental Table 1). Other investigators have similarly observed an association between base of skull [32, 33] and optic nerve injuries with injury severity and in hospital mortality following trauma [30, 33]. Some investigators have extended cranial nerve evaluations to include the TBI with oculomotor, trochlear and abducens nerves. We observed only 145 cases for all these injuries and elected not to separate them for separate analysis. However, Jin et al. [34] showed an association between cranial nerve injury associated with TBI and high morbidity and mortality rates. Indeed, isolated third cranial nerve palsy has been associated with a poor post-traumatic prognosis [35]. Future studies with larger databases may corroborate these findings, however, it is unlikely that they will surpass the impact of traumatic optic neuropathy on morbidity and mortality.

### Mechanisms and intentions

Our study confirmed previously reported findings that firearms and self-inflicted injuries were associated with more severe injury indices and thus mortality [19, 36, 37]. Cut/piece injuries have lower severity indices and lower mortality. In a study of all patients admitted with major trauma and ocular injuries, He et al. [17] found that victims of firearms injuries were 3.73 times greater odds of very severe ISS and 4.77 times greater odds of severe TBI than other mechanisms of injury. Compared to a 3.9% mortality for all admitted patients with ocular trauma, victims of firearms injuries had a mortality rate of 16.2% [17, 36]. In this study all 1409 patients admitted with firearms -related were documented to have TBI. Most firearms-related ocular injury reports have been studies of non-powder guns [38, 39]. There’s a dearth of reports that include powder firearms -related ocular trauma that report mortality. However, a study conducted at one urban center found the mortality was 18%. Our findings that used the same data source as Truong et al., comport with these findings of high mortality [40]. General trauma literature has similarly found higher severity indices and mortality for firearms injury [41, 42] and a propensity for self-inflicted firearms injuries to involve the head and face [41, 43]. A recent study used CDC data and heat maps from 1990 to 2021 to evaluate trends in demographic and regional disparities in firearms-related mortality [44]. They found demographic disparities that mirror our findings with similarly disproportionate representation of male and Black patients. Assault (homicide) was most common amongst Black and Hispanic patients and suicide amongst white patients. Additionally, the noted a disturbing trend of increasing firearm-related suicides amongst White non-Hispanic females and firearm-related assault deaths amongst Hispanic and Black non-Hispanic females [44]. 

Firearm-related deaths are a major growing public health issue in the United States with a total annual cost of $557 billion (2.6% of GDP) [45]. A recent Pew Research Center report [46] collated data from sources including the CDC and FBI (Federal Bureau of Investigation) to evaluate trends in gun violence in the US. They found that the annual rate of firearms-related deaths in 2021 was 48,830, an increase of 23% since the 2019 (pre-COVID pandemic) rate. Incidents of mass-shootings increased by 51% in the same period. This report also noted that most injuries, 54%, were due to suicide, followed by 43% due to assault or homicide. The remainder comprised unintentional, law enforcement and undetermined intentions [46]. More than half of suicides, 55%, involved a firearm. Firearm-related injuries now exceed motor vehicle accidents as the leading cause of premature death from trauma in individuals less than 24years of age [16, 47, 48]. This is thought to have resulted from simultaneous rise in gun violence and decrease in motor vehicle accidents. Although attention is directed at mortality rates from these injuries, the vast majority survive to live with various degrees of disability. Physicians need to be vigilant and triage and manage patients appropriately to improve survival rates in patients admitted with major trauma associated with firearms.

Similarly lacking are reports focused on intentions and ocular injuries. Gise et al. [30] in an assessment of intentions in pediatric ocular injuries found that victims of self-inflicted injuries were most likely to have TBI, very severe ISS and severe GCS. These children had 8.5 times higher odds of mortality than other intentions. This study found that firearms were the most likely mechanism [19]. Other trauma investigators have reported similarly high injury severity and mortality rates with self-inflicted trauma than other intentions [41–43]. 

### Demographic differences

Demographic differences were observed regarding mechanisms and intention of mortality-related injuries. In general, we affirmed previous study observations with higher rates amongst male, white, and Southern populations. Black patients were most likely to be victims of assault-related ocular trauma whereas White patients were most likely to have fall-related injuries. Hispanic and Asians were most likely victims of MVT-pedestrian injuries. Although more White patients expired overall, Black patients were almost twice as likely to expire following severe injury (GCS < 8, ISS > 24). Other minorities (Asians, Hispanics) were also more likely to expire, but not as noteworthy when compared to White patients. Interestingly, Black and Hispanic were victims to different mechanisms of injury, however, were similarly most likely to die within the first day of admission.

Black patients have the highest odds of firearm-related injury than other races/ethnicities and such injuries are associated with high rates of mortality [49, 50]. However, it does not explain the two-fold difference in likelihood of death between White and Black patients at the same level of injury severity score and TBI at presentation and higher likelihood of death within one day of admission. Improved understanding of factors that contribute to fatality associated with major trauma with ocular injuries can promote physician surveillance of patients that present with high-risk features. Furthermore, results can guide development of public health interventions that mitigate primary risk in the general population.

Several studies have elucidated the role of race in disease management. Two studies that utilized the NTDB, from an earlier time, found that minority patients with severe TBI were less likely to be placed in rehabilitation compared to their White counterparts after acute hospitalization [51, 52]. Owens et al. [53] found that in ED settings, racial and ethnic minorities had lower prioritization due to lower triage scores, experienced longer wait times, and were more likely to leave the ED without being seen by a provider. These studies show that prior to and following hospitalization, there are race-based biases in treatment practices. The same could hold true during in-patient management and is one such explanation for the findings in this study. Regardless, it is important to raise awareness of this issue amongst clinicians to ensure more equitable evaluation and management [54]. A recently published report of firearms-related ocular trauma found that race/ethnicity disparities in care extended to discharge priorities. Black and Hispanic patients were less likely to be allocated to advanced care facility than their White counterparts. This was noted for all insurance coverage and injury severity levels [55]. Further research that illuminates specific factors associated with increased mortality of minority patients admitted with very severe injury is warranted. A deeper inspection of implicit bias in global healthcare delivery and education of caregivers will help to prevent unnecessary trauma-related deaths.

### Insurance coverage

Interestingly, we also found that insurance coverage was associated with mortality. Those with commercial insurance were more likely to survive their injuries than those with government insurance (Medicare/Medicaid) and self-paying patients. Previous studies have delineated risk factors leading to ocular trauma, including age < 30 years, male gender, and lower socioeconomic status [56–58]. In our cohort of 316,485 patients who suffered ocular trauma, 3.86% succumbed to their injuries. Patients most likely to expire were ≥ 65 years old, male, white, and of lower socioeconomic status (SES). Although we defined those with Medicare insurance as being of lower SES, our estimations may be confounded by the fact that those entitled to this insurance usually are ≥ 65year old. Insurance is often an indicator of SES, and others have found that public insurance (Medicare and Medicaid) holders are generally of a lower SES than commercial insurance holders [59]. 

Low et al. [60] showed that lower socioeconomic status (SES) is correlated to higher incidence of severe ocular trauma. Patients who are uninsured or have public health insurance have been shown to have poorer health outcomes compared to patients with private health insurance [61]. Our study affirms these findings regarding trauma-related mortality. In this population, patients with Medicare had the greatest odds of mortality while patients with commercial insurance had the greatest odds of survival. This may be attributed to several factors: patients with lower SES are more likely to suffer from chronic conditions, [62–64] have limited healthcare literacy, [65] and decreased access to healthcare resources [66]. Poor initial health status place individuals at a lower baseline health prior to the trauma itself and has more devastating impact on outcomes [67–69]. 

### Limitations

This study is limited by the breadth and depth of data accessed from the NTDB. Data was accessed from 2008 to 2014 and accuracy of the results is dependent upon proper documentation and ICD-9 CM coding by submitting teams. For analytic expedience, we categorized injury severity based on ISS, GCS, and presence of TBI; we did not detail the specifics of extracranial and non-ocular injuries. While injury may have had concomitant cranial and ocular injuries, the primary injury that ultimately led to demise was not captured in this data analysis. In addition, when calculation of odds ratios, can only determine the strength of association of variables but cannot draw conclusions about causal relations. Lastly, NTDB does not detail ophthalmic examinations. Thus, our study did not address visual outcomes prior to mortality, which is another factor in characterizing the extent of ocular injury. Documentation is based on ED/Trauma team assessment and may not reflect subsequent Ophthalmic assessments. An example of the ocular injury category of “retinal edema,” it is not clear whether this means OCT-determined retinal edema or commotio retinae. Furthermore, a full detailing of all ophthalmic injuries that would require slit lamp examination, and ocular motor (III, IV, VI) and ancillary visual field and optic coherence tomography assessment may not be possible in the context of major trauma. Thus, the estimation of various ophthalmic lesions within this database may represent underestimations. Despite these limitations, this study has merits of using a large trauma database to determine factors associated with mortality in patients admitted with major trauma and ocular injuries. Although providing a foundation, future studies are required to confirm our findings. Use of up-to-date data from 2014 to present day with use of current ICD10 codes, with more consideration of whole-body injuries and associated visual outcomes prior to mortality would add to our knowledge base.

## Conclusions

In this study of the NTDB (2008–2014), non-fatal and mortality-related ocular trauma had differences in mechanism of injury, age, gender, race, and injury severity. Male, White, ≥ 65 years old and, Medicare-covered patients were most likely be victims of mortality-related trauma. Injuries resulting in very severe injury severity scores and severe traumatic brain injury (GCS) had the highest association with mortality. Firearms were the deadliest mechanism and the most likely to cause death in the elderly and within a day of admission. Cut/pierce injury was the most benign mechanism and most likely to be survived by the young. Black patients admitted with high injury severity scores were more likely to die than other race/ethnic groups in the same severity categories. To our knowledge, our investigation is the first to characterize risk factors of major trauma and ocular injury associated with mortality. Observed factors may be useful in identifying at risk individuals and help in developing focused public health measures aimed at preventing trauma-related deaths.

### Electronic supplementary material

Below is the link to the electronic supplementary material.

Supplementary Material 1

## Data Availability

The datasets generated and/or analyzed during the current study are available from the NTDB® website: https://www.facs.org/quality-programs/trauma/quality/national-trauma-data-bank/.

## Declarations

### Meeting presentations

1. Characteristics of Ocular Injuries Associated with Mortality in Patients Admitted with Major Trauma, AAO, San Francisco, Ca, October 2019.

2. Comparative Analysis of Mechanisms Associated with Non-Fatal and Mortality Related Ocular Trauma, WOC, Cape Town (Virtual), June 2020.
