# Supplementary material for: Characteristics of ocular injuries associated with mortality in patients admitted with major trauma
Source: BMC Ophthalmol. 2024 Mar 19;24:125. doi: 10.1186/s12886-024-03392-y (PMC10949718; doi:10.1186/s12886-024-03392-y)
Supplement: Supplementary file 1 — Supplementary Material 1 [file 12886_2024_3392_MOESM1_ESM.docx]

**SUPPLEMENTAL DIGITAL CONTENT**

**Supplemental Table 1a:** Simple Logistic Regression Analysis of Head Injuries and Mortality in Survivors, NTDB 2008-2014

|  | **Head Injury (Total)** | **Frequency (% of total)** | **P-value** | **Odds Ratio** | **95% Confidence Interval** |
| --- | --- | --- | --- | --- | --- |
| Survivors | Extradural Hemorrhage (n=4401) | 3986 (90.6%) | <0.001 | .378 | .341-0.419 |
|  | Subarachnoid Hemorrhage  (n=32766) | 28655 (87.5%) | <0.001 | .205 | 0.197-0.214 |
|  | Subdural Hemorrhage (n=32632) | 28301 (86.7%) | <0.001 | .187 | 0.180-0.195 |
|  | Cerebral Contusion (n=17079) | 15121 (88.5%) | <0.001 | .274 | 0.261-0.289 |
|  | Intracerebral Hemorrhage (n=18451) | 15289 (82.9%) | <0.001 | .152 | 0145-0.159 |
|  | Vault Skull Fracture (n=28166) | 24788 (88.0%) | <0.001 | .233 | 0.223-0.242 |
|  | Base of Skull Fracture (n=19342) | 16324 (84.4%) | <0.001 | .173 | 0.166-0.181 |
|  | Fracture of Face Bone (n=182279) | 174786 (95.9%) | <0.001 | .854 | 0.823-0.886 |

**Supplemental Table 1b:** Simple Logistic Regression Analysis of Head Injuries and Mortality in Expired, NTDB 2008-2014

| Expired | Extradural Hemorrhage (n=4401) | 415 (9.4%) | <0.001 | 2.645 | 2.387-2.932 |
| --- | --- | --- | --- | --- | --- |
|  | Subarachnoid Hemorrhage  (n=32766) | 4111 (12.5%) | <0.001 | 4.868 | 4.680-5.064 |
|  | Subdural Hemorrhage (n=32632) | 4331 (13.3%) | <0.001 | 5.344 | 5.140-5.557 |
|  | Cerebral Contusion (n=17079) | 1958 (11.5%) | <0.001 | 3.644 | 3.462-3.834 |
|  | Intracerebral Hemorrhage (n=18451) | 3162 (17.1%) | <0.001 | 6.588 | 6.307-6.882 |
|  | Vault Skull Fracture (n=28166) | 3378 (12.0%) | <0.001 | 4.301 | 4.125-4.484 |
|  | Base of Skull Fracture (n=19342) | 3018 (15.6%) | <0.001 | 5.777 | 5.528-6.037 |
|  | Fracture of Face Bone (n=182279) | 7493 (4.1%) | <0.001 | 1.171 | 1.128-1.215 |

**Supplemental Table 2:** Simple Logistic Regression Analysis of Intent of Injury in Expired Patients by Age, Gender, and Race/Ethnicity, NTDB 2008-2014



|  | **Category** | | **Intent** | **Frequency (% of total)** | **P-value** | **Odds Ratio** | **95% Confidence Interval** |
| --- | --- | --- | --- | --- | --- | --- | --- |
| Expired | Age | <20 | Assault | 633 (42.86) | <0.001 | 6.539 | 5.777-7.401 |
|  |  |  | Self-Inflicted | 87 (9.81) | <0.001 | 0.617 | 0.486-0.777 |
|  |  |  | Unintentional | 884 (10.1) | <0.001 | 0.260 | 0.233-0.291 |
|  |  | 21-64 | Assault | 732 (49.56) | 0.014 | 1.147 | 1.027-1.282 |
|  |  |  | Self-Inflicted | 595 (67.08) | <0.001 | 2.508 | 2.163-2.908 |
|  |  |  | Unintentional | 3,834 (43.85) | <0.001 | 0.610 | 0.557-0.667 |
|  |  | >65 | Assault | 81 (5.48) | <0.001 | 0.094 | 0.074-0.119 |
|  |  |  | Self-Inflicted | 161 (18.15) | <0.001 | 0.409 | 0.342-0.489 |
|  |  |  | Unintentional | 3,542 (40.51) | <0.001 | 5.891 | 5.133-6.763 |
|  | Gender | Male | Assault | 1095 (73.69) | <0.001 | 1.244 | 1.098-1.410 |
|  |  |  | Self-Inflicted | 766 (85.40) | <0.001 | 2.687 | 2.214-3.263 |
|  |  |  | Unintentional | 6,270 (67.55) | <0.001 | 0.585 | 0.526-0.650 |
|  |  | Female | Assault | 391 (26.31) | <0.001 | 0.804 | 0.709-0.910 |
|  |  |  | Self-Inflicted | 131 (14.60) | <0.001 | 0.372 | 0.306-0.452 |
|  |  |  | Unintentional | 3,012 (32.45) | <0.001 | 1.710 | 1.540-1.900 |
|  | Race/Ethnicity | White | Assault | 633 (42.6) | <0.001 | 0.229 | 0.205-0.257 |
|  |  |  | Self-Inflicted | 739 (82.39) | <0.001 | 1.882 | 1.572-2.255 |
|  |  |  | Unintentional | 7,055 (76.01) | <0.001 | 2.301 | 2.095-2.526 |
|  |  | Black | Assault | 466 (31.36) | <0.001 | 5.414 | 4.738-6.186 |
|  |  |  | Self-Inflicted | 41 (4.57) | <0.001 | 0.378 | 0.267-0.520 |
|  |  |  | Unintentional | 741 (7.98) | <0.001 | 0.328 | 0.290-0.371 |
|  |  | Hispanic | Assault | 272 (18.30) | <0.001 | 2.291 | 1.971-2.664 |
|  |  |  | Self-Inflicted | 76 (8.47) | 0.094 | 0.813 | 0.629-1.039 |
|  |  |  | Unintentional | 825 (8.89) | <0.001 | 0.575 | 0.503-0.657 |
|  |  | Asian | Assault | 26 (1.75) | 0.253 | 0.788 | 0.502-1.190 |
|  |  |  | Self-Inflicted | 16 (1.78) | 0.429 | 0.814 | 0.456-0.814 |
|  |  |  | Unintentional | 208 (2.24) | 0.201 | 1.234 | 0.890-1.743 |

**Supplemental Table 3a.** Simple Logistic Regression Analysis of Severe Glasgow Coma Scale (GCS) ≤8 in Survivors by Age, Gender, and Race/Ethnicity, NTDB 2008-2014

|  | **Glasgow Coma Scale (GCS)** | **Category** | | **Frequency**  **(% of total)** | **P-value** | **Odds Ratio** | **95% Confidence Interval** |
| --- | --- | --- | --- | --- | --- | --- | --- |
| **Survivors** | GCS ≤8 (Severe TBI)* | Age | <20  (n=50760) | 6562 (12.9%) | <0.001 | 0.007 | 0.006-0.009 |
|  |  |  | 21-64  (n=17074) | 22162  (13.0%) | <0.001 | 0.028 | 0.026-0.030 |
|  |  |  | ≥65  (n=48670) | 2446  5.0% | <0.001 | 0.061 | 0.056-0.065 |
|  |  | Gender | Male  (n=191619) | 23993  (12.5%) | <0.001 | 0.049 | 0.047-0.052 |
|  |  |  | Female  (n=84472) | 7368  (8.7%) | <0.001 | 0.056 | 0.052-0.060 |
|  |  | Race/Ethnicity | White  (n=181799) | 20572  (11.3%) | <0.001 | 0.059 | 0.056-0.062 |
|  |  |  | Black  (n=42220) | 4378  (10.4%) | <0.001 | 0.031 | 0.027-0.036 |
|  |  |  | Hispanic  (n=34526) | 4044  (11.7%) | <0.001 | 0.032 | 0.028-0.037 |
|  |  |  | Asian  (n=4678) | 484  (10.3%) | <0.001 | .052 | .039-0.069 |

**Supplemental Table 3b.** Simple Logistic Regression Analysis of Severe Glasgow Coma Scale (GCS) ≤8 in Expired by Age, Gender, and Race/Ethnicity Gender, NTDB 2008-2014

|  | **Glasgow Coma Scale (GCS)** | **Category** | | **Frequency (% of total)** | **P-value** | **Odds Ratio** | **95% Confidence Interval** |
| --- | --- | --- | --- | --- | --- | --- | --- |
| **Expired** | GCS ≤8 (Severe TBI) * | Age | <20  (n=1560) | 1486  (95.3%) | <0.001 | 135.255 | 106.941-171.065 |
|  |  |  | 21-64  (n=5770) | 4850  84.1% | <0.001 | 35.344 | 32.893-37.978 |
|  |  |  | ≥65  (n=3598) | 1677  46.6% | <0.001 | 16.497 | 15.273-17.820 |
|  |  | Gender | Male  (n=8083) | 6016  (74.4%) | <0.001 | 20.334 | 19.308-21.415 |
|  |  |  | Female  (n=3385) | 2140  (63.2%) | <0.001 | 17.988 | 16.707-19.366 |
|  |  | Race/Ethnicity | White  (n=5652) | 2597  (31.5%) | <0.001 | 17.057 | 16.246-17.907 |
|  |  |  | Black  (n=1238) | 974  (78.7%) | <0.001 | 31.890 | 27.736-36.666 |
|  |  |  | Hispanic  (n=964) | 234 (19.5%) | <0.001 | 31.052 | 26.819-35.953 |
|  |  |  | Asian  (n=249) | 172  (69.1%) | <0.001 | 19.398 | 14.591-25.788 |

***TBI= traumatic brain injury**

**Supplemental Table 4a.** Simple Logistic Regression Analysis of Very Severe Injury Severity Score (ISS) >24 in Survivors by Age, Gender, and Race/Ethnicity, NTDB 2008-2014

|  | **Injury Severity Score (ISS)** | **Category** | | **Frequency**  **(% of total)** | **P-value** | **Odds Ratio** | **95% Confidence Interval** |
| --- | --- | --- | --- | --- | --- | --- | --- |
| **Survivors** | ISS >24 | Age | <20  (n=54743) | 6256  (11.4%) | <0.001 | .024 | .021-0.027 |
|  |  |  | 21-64  (n=177964) | 21331  (12.0%) | <0.001 | .033 | .031-0.035 |
|  |  |  | ≥65  (n=52219) | 4480  (8.6%) | <0.001 | .079 | .074-0.085 |
|  |  | Gender | Male  (n=201459) | 23498  (11.7%) | <0.001 | .048 | .046-0.051 |
|  |  |  | Female  (n=89605) | 8938  (10.0%) | <0.001 | .061 | .056-0.065 |
|  |  | Race/Ethnicity | White  (n=195148) | 22452  (11.5%) | <0.001 | .059 | .056-0.062 |
|  |  |  | Black  (n=44508) | 3800  (8.5%) | <0.001 | .031 | .027-0.035 |
|  |  |  | Hispanic  (n=33597) | 3654  (10.9%) | <0.001 | .033 | .028-0.038 |
|  |  |  | Asian  (n=4470) | 607  13.6% | <0.001 | .043 | .031-0.060 |

**Supplemental Table 4b:** Simple Logistic Regression Analysis of Very Severe Injury Severity Score (ISS) >24 in Expired by Age, Gender, and Race/Ethnicity, NTDB 2008-2014

|  | **Injury Severity Score (ISS**) | **Category** | | **Frequency**  **(% of total)** | **P-value** | **Odds Ratio** | **95% Confidence Interval** |
| --- | --- | --- | --- | --- | --- | --- | --- |
| **Expired** | ISS >24 | Age | <20  (n=1616) | 1365  (84.5%) | <0.001 | 42.149 | 36.747-48.345 |
|  |  |  | 21-64  (n=5793) | 4662  (80.5%) | <0.001 | 30.268 | 28.320-32.350 |
|  |  |  | ≥65  (n=3680) | 1996  (54.2%) | <0.001 | 12.630 | 11.756-13.569 |
|  |  | Gender | Male  (n=8143) | 5964  (73.2%) | <0.001 | 20.729 | 19.700-21.811 |
|  |  |  | Female  (n=3474) | 2244  (64.6%) | <0.001 | 16.465 | 15.308-17.710 |
|  |  | Race/Ethnicity | White  (n=8488) | 5833  68.7% | <0.001 | 16.899 | 16.108-17.729 |
|  |  |  | Black  (n=1260) | 945  75.0% | <0.001 | 32.138 | 28.170-36.665 |
|  |  |  | Hispanic  (n=1089) | 859  78.9% | <0.001 | 30.605 | 26.355-35.541 |
|  |  |  | Asian  (n=224) | 176  78.6% | <0.001 | 23.335 | 16.769-32.472 |
